# Narrative literature review of antidiabetic drugs’ effect on hyperuricemia: elaborating actual data and mechanisms

**DOI:** 10.1530/EC-24-0070

**Published:** 2024-05-10

**Authors:** Zhenyu Liu, Huixi Kong, Baoyu Zhang

**Affiliations:** 1 Department of Clinical Medicine, Beijing Luhe Hospital, Capital Medical University, Tongzhou District, Beijing, China; 2 Department of Clinical Medicine, Beijing Shijitan Hospital, Capital Medical University, Haidian District, Beijing, China; 3 Center for Endocrine Metabolism and Immune Diseases, Beijing Luhe Hospital, Capital Medical University, Tongzhou District, Beijing, China

**Keywords:** antidiabetic drugs, hyperuricemia, serum uric acid, type 2 diabetes mellitus

## Abstract

To optimize the treatment plan for patients with type 2 diabetes mellitus (T2DM) and hyperuricemia, this narrative literature review summarizes the effect of antidiabetic drugs on serum uric acid (SUA) levels using data from observational studies, prospective clinical trials, *post* *hoc* analyses, and meta-analyses. SUA is an independent risk factor for T2DM, and evidence has shown that patients with both gout and T2DM exhibit a mutually interdependent effect on higher incidences. We find that insulin and dipeptidyl peptidase 4 inhibitor (DPP-4i) except linagliptin could increase the SUA and other drugs including metformin, thiazolidinediones (TZDs), glucagon-like peptide-1 receptor agonists (GLP-1 RAs), linagliptin, sodium–glucose cotransporter 2 inhibitors (SGLT2i), and α-glucosidase inhibitors have a reduction effect on SUA. We explain the mechanisms of different antidiabetic drugs above on SUA and analyze them compared with actual data. For sulfonylureas, meglitinides, and amylin analogs, the underlying mechanism remains unclear. We think the usage of linagliptin and SGLT2i is the most potentially effective treatment of patients with T2DM and hyperuricemia currently. Our review is a comprehensive summary of the effects of antidiabetic drugs on SUA, which includes actual data, the mechanisms of SUA regulation, and the usage rate of drugs.

## Introduction

According to recent statistics, 529 million people will have diabetes worldwide in 2021, and the global age-standardized total diabetes prevalence will be 6.1% (1). Type 2 diabetes mellitus (T2DM) is the most frequent form of diabetes, accounting for 90–95% of all patients with diabetes (2), and the number of patients with T2DM is predicted to reach 439 million by 2030 (3). Based on the National Health and Nutrition Examination Survey (NHANES), a survey estimated mean serum uric acid (SUA) levels as 6.0 mg/dL in men and 4.8 mg/dL in women. Hyperuricemia was diagnosed according to the clinical diagnostic criteria, and the SUA level cutoff was 420 μmol/L for males and 360 μmol/L for females (4), and hyperuricemia prevalence rates were 20.2% and 20.0% in men and women, respectively (5). In China, the overall prevalence of hyperuricemia was 11.1% in 2015–2016 and 14.0% in 2018–2019, reflecting an alarming 3-year increase (6). Similar trends have also been observed in many other countries (7, 8). The combined prevalence of diabetes among patients with hyperuricemia and gout was 19.00% and 17.80%, respectively (9). A cross-sectional survey reported that SUA of patients with diabetes was 344.05 ± 90.89 μmol/L (10). Moreover, several studies have reported an association between hyperuricemia and adverse cardiovascular outcomes. Two of the main effects of hypertension, stroke and heart failure, have been linked to elevated SUA levels (11, 12, 13). A recent investigation confirmed that SUA levels are an independent predictor of cardiovascular mortality (14). Diabetes-related complications, particularly cardiovascular diseases (CVDs), are the leading causes of morbidity and mortality among patients with T2DM (15, 16). Thus, to optimize the treatment plan, antidiabetic drugs that can reduce SUA levels are required to achieve comprehensive control in patients with T2DM and hyperuricemia. Therefore, this review aimed to summarize how antidiabetic drugs affect SUA levels and to recommend drug use for patients with T2DM.

## Effect of antidiabetic drugs on SUA levels

Antidiabetic drugs can be classified into the following two groups based on their distinct effects in decreasing blood sugar: those that increase insulin secretion and those that rely on other mechanisms. The former drugs mainly include sulfonylureas, meglitinides, dipeptidyl peptidase-4 inhibitors (DPP-4i), whereas the latter drugs that lower blood sugar through other mechanisms mainly include metformin, thiazolidinediones (TZDs) α-glucosidase inhibitors, and sodium–glucose cotransporter 2 inhibitors (SGLT2i). The formation of serum uric acid (SUA) is the ribose 5-phosphate, a pentose derived from glycidic metabolism, converted to phosphoribosyl pyrophosphate (PRPP) and then to phosphoribosyl amine, that will be transformed into inosine monophosphate (IMP). From this intermediate compound derive adenosine monophosphate (AMP), guanosine monophosphate (GMP), and inosine which will be degraded into hypoxanthine and xanthine and finally into SUA. (17) SUA exists majorly as urate, so humans cannot oxidize SUA to the more soluble compound allantoin due to the lack of uricase enzyme. Normally, most daily SUA degradation occurs via the kidneys (18). The kidneys eliminate approximately two-thirds, while the gastrointestinal tract eliminates one-third of the uric acid load. Almost all uric acid is filtered from glomeruli, while post-glomerular reabsorption and secretion regulate the amount of uric acid excretion. The proximal tubule is the site of uric acid reabsorption and secretion, and approximately 90% is reabsorbed into blood (19). Therefore, we analyzed the overall pharmacological effects from the actual data and the specific mechanisms provided ahead.

### Insulin

**Figure 1:**
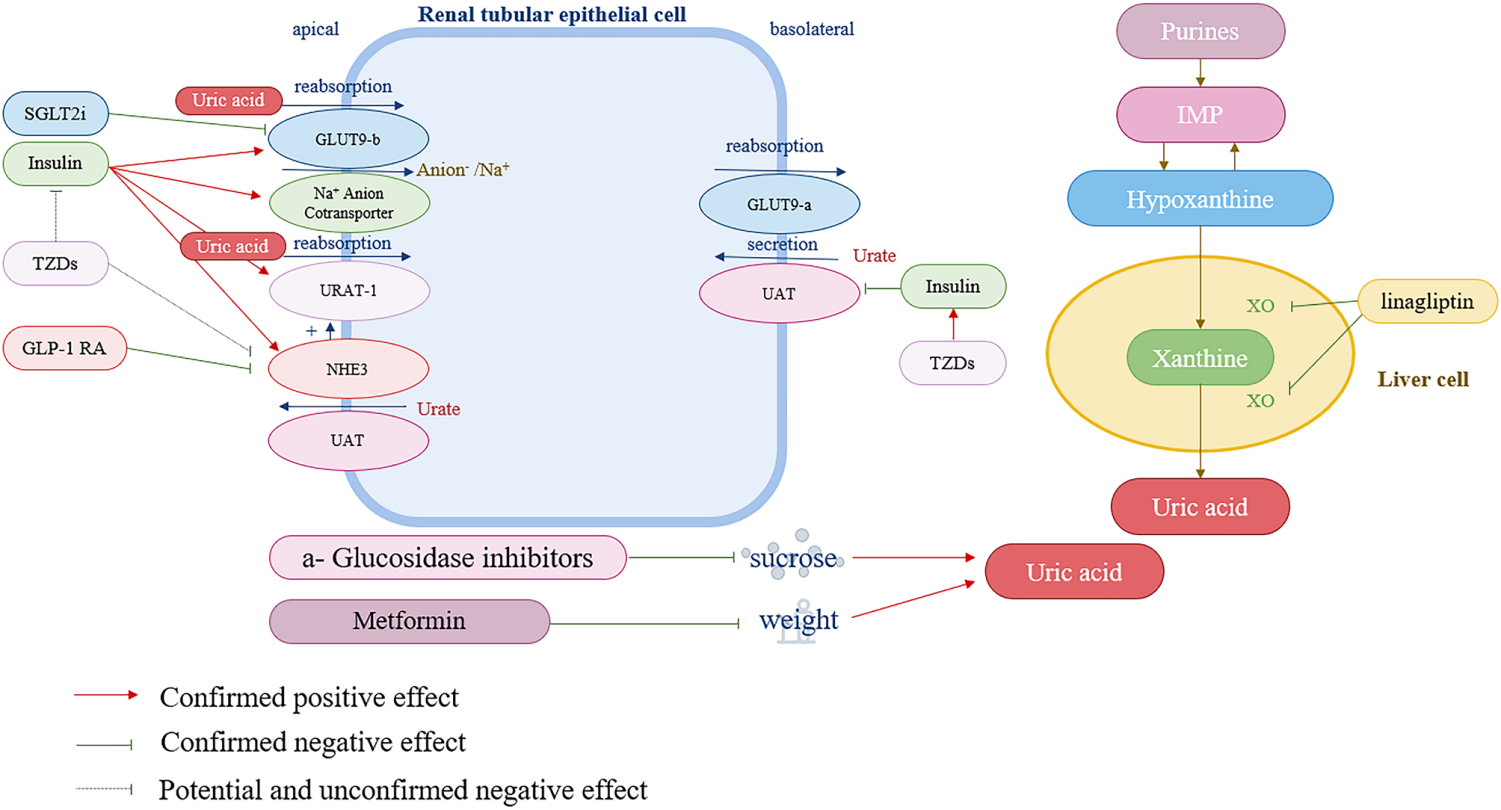
The mechanisms of the effect of antidiabetic drugs on SUA.

Type 1 diabetes mellitus (T1DM) is an endocrine disorder in which pancreatic β cells do not secrete insulin, typically due to autoimmune destruction. Thus, insulin replacement is vital for its management (12). In T2DM, despite the availability of oral glucose-lowering drugs, insulin supplementation is often required to achieve favorable glucose control (13). A matched-cohort study (20) (*n* = 223 patients with gout and DM) showed that insulin therapy led to a significant mean increase in SUA levels (Table 1). Meanwhile, another investigation found that the baseline hyperinsulinemia follow-up hyperuricemia group had the greatest incidence risk of T2DM (27.9%) and that the follow-up SUA level had a 5.5% mediation effect on the insulin–T2DM connection. This phenomenon may be increased by renal urate reabsorption via the stimulation of GLUT9 (21) (encoded bySLC2A9), urate transporter 1 (URAT-1), and the sodium-dependent anion cotransporter in the proximal tubule (22) (Fig. 1). Furthermore, acute hyperinsulinemia during insulin clamp significantly decreases the urinary excretion of uric acid (UE_UA_) (23). In conclusion, hyperinsulinemia causes hyperuricemia and insulin could increase SUA levels.

**Table 1:** Effect of antidiabetic drugs on SUA.

Antidiabetic drug	Specific drug types	SUA	Clinical data	Participants (number)	Design	Reference
Insulin	–	↑	Δ 1.25 mg/dL	23 patients with gout and DM	Matched cohort study	(20)
Metformin	–	↑	Δ 0.7 ± 1.1 mg/dL	16 patients with T2DM	RCT	(25)
		↓	–	76 patients with T2DM	Before–after study	(26)
		↑ ⇔ ↓	3 of them increased 11 of them remained 12 of them decreased	26 patients with gout	Before–after study	(27)
TZDs	Pioglitazone	⇔	Those SUA >6.0 mg/dL decreased those 3.8 < SUA < 5.9 mg/dL remained	68 patients with T2DM	A cohort study	(35)
		⇔	–	36 patients with idiopathic nephrolithiasis	RCT	(36)
	Rosiglitazone	↓	Δ 0.4 ± 1.4 mg/dL	16 patients with T2DM	RCT	(25)
GLP-1 RA	Exenatide	⇔	–	36 patients with T2DM and overweight	*Post* *hoc* analysis of a randomized, open-label, active-comparator, parallel-group trial	(40)
	Exenatide lixisenatide liraglutide	⇔	–	Study A: 9 healthy men with overweight Study B: 52 patients with overweight and T2DM Study C: 36 patients with overweight and T2DM Study D: 35 patients with overweight and T2DM	*Post* *hoc* analyses of four clinical trials	(41)
	–	↓	Δ 0.34 mg/dL	20 studies	Meta-analysis	(42)
DPP-4i	Sitagliptin	↑	From 5.07 ± 1.21 to 5.40 ± 1.45 mg/dL	940 patients with T2DM	Retrospective, observational study	(46)
		↑	From 5.08 ± 1.14 to 5.30 ± 1.24 mg/dL	120 patients with T2DM	Multicenter, randomized, open-label, parallel-group trial	(47)
		↑	From 4.91 ± 1.28 to 5.42 ± 1.43 mg/dL	64 patients with T2DM	Prospective, nonrandomized, observational study	(48)
		↑	Δ 0.3 ± 0.1 mg/dL	163 patients with T2DM	Prospective, randomized, open-label, blinded-endpoint, parallel-group trial	(55)
	Alogliptin	↑	From 4.69 ± 1.60 to 5.24 ± 1.61 mg/dL	55 patients with T2DM	Prospective, nonrandomized, observational study	(48)
	Linagliptin	↓	From 5.63 ± 1.24 to 5.24 ± 1.10 mg/dL	73 patients with T2DM	Before–after study	(49)
SGLT2i	Empagliflozin	↓	Δ 36.59 μmol/L for 10 mg Δ 43.55 μmol/L for 25 mg	12 RCTs including 5781 patients with T2DM	Meta-analysis	(53)
	Dapagliflozin	↓	Δ 68.03 μmol/L	experiments:29 patients with T2DM controls: 30 patients with T2DM	RCT	(54)
		↓	The dapagliflozin group: Δ 0.5 ± 0.1 mg/dL	168 patients with T2DM	Prospective, randomized, open-label, blinded-endpoint, parallel-group trial	(55)
	Canagliflozin	↓ ⇔	Overall: from 5.50 ± 1.21 to 5.25 ± 1.19 mg/dL Group lost: from 5.75 ± 1.05 to 5.35 ± 1.17 mg/dL Group neutral: remained	Group lost: 20 patients with T2DM and weight-loss Group neutral: 16 patients with T2DM and no weight-loss	*Posthoc* analysis	(58)
		↓	Overall: from 5.29 ± 1.25 to 5.16 ± 1.20 mg/dL Group A: from 4.79 ± 1.22 to 5.34 ± 1.39 mg/dL Group B: from 5.80 ± 1.08 to 4.98 ± 0.97 mg/dL	Group A: 20 patients with T2DM and higher baseline SUA Group B: 20 patients with T2DM and lower baseline SUA	Before–after study	(59)
α-Glucosidase inhibitors	Acarbose	↓	sucrose: 4.9 ± 1.0 to 5.4 ± 1.1 mg/dL sucrose + acarbose: 4.7 ± 1.3 to 4.9 ± 1.4 mg/dL	6 healthy subjects	Clinical trial	(68)
Sulfonylureas	Gliclazide	⇔	–	29 patients with T2DM	Before–after study	(70)

RCT, randomized controlled trial.

### Metformin

Metformin is the most widely used and intensively studied drug recommended as a first-line therapy, accounting for 55.6% of patients with T2DM worldwide (24). A survey of the treatment patterns of oral drug users in China shows that 53.7% of patients with T2DM used metformin. As shown by an investigation, SUA levels were higher in experiments (*n* = 64 patients with T2DM) than those in controls (*n* = 47 patients with T2DM). Similarly, SUA levels increased after 18 weeks of metformin treatment (*n* = 16 patients with T2DM) (Table 1) (25). Nevertheless, a 1996 trial (*n* = 76 patients with T2DM) demonstrated that adding low-dose metformin to sulfonylurea treatment led to significant improvements in blood lipids and blood sugar, as well as a considerable reduction in SUA and body weight (26). In another trial, 26 patients with gout and insulin resistance (IR) were treated with metformin. Follow-up after 6 months later revealed that 12 patients had significantly lower SUA levels and 11 had normal SUA levels (27). The same result was reported in that the incidence of hyperuricemia significantly decreased (*n* = 10 patients with T2DM) although SUA levels did not significantly decrease (28). In addition to reducing SUA levels through weight loss (Fig. 1), metformin may also have a notable impact on appetite. This resulted in a low-purine diet and helped control SUA levels. More importantly, by reducing IR, which minimizes the risk of gout (29), metformin may lower SUA synthesis, and increase SUA excretion. Metformin also promoted the phosphorylation of AMP-activated protein kinase (AMPK) and restored insulin-stimulated glucose uptake in hyperuricemia-induced IR cardiomyocytes. It could delay the development of atherosclerosis and inflammation through this effect (30). Metformin enhances IR and glucose tolerance in a rat model of acute hyperuricemia. Additionally, it is associated with the increased phosphorylation of protein kinase B, AMPK, and GLUT4 in the myocardial tissues (31, 32). Moreover, some studies have indicated that a reduction in BMI, SUA levels at baseline, and metformin therapy are all independent predictors of a decline in SUA levels. However, the physiological mechanisms that affect SUA levels remain unclear.

### Thiazolidinediones

The guideline indicated (34) that thiazolidinediones (TZDs) mainly reduce blood sugar by increasing target cell sensitivity. Most likely because of safety issues, use of TZDs has decreased (34). Pioglitazone and rosiglitazone are the two main TZDs. After 12 weeks of pioglitazone treatment (*n* = 68 patients with T2DM), SUA changes depended on baseline SUA levels. In particular, the higher the baseline SUA level, the greater the decrease. When the baseline SUA level was greater than 6.0 mg/dL, the SUA levels decreased (Table 1), but did not change in individuals whose baseline SUA was between 3.8 and 5.9 mg/dL (35). Notably, 24-week pioglitazone treatment reduced the acid load in the kidneys of 36 patients with idiopathic uric acid nephrolithiasis, resulting in a significantly greater urine pH (36). This leads to enhanced urinary uric acid excretion and, consequently, reduced SUA levels. Similar results have been reported previously (36). Rosiglitazone treatment (*n* = 16 patients with T2DM) had similar effects (25). Animal experiments have shown that TZD drugs indirectly regulate the expression of UAT and URAT1 mRNA by improving IR and reducing blood insulin levels, thereby reducing hyperuricemia caused by hyperinsulinemia. In rats, the brush-border membrane Na+/H+ exchanger 3 (NHE3) had lower expression and activity, which is the principal mediator of proximal tubule ammonium excretion, had lower expression and activity (37). NHE3 increases SGLT2 expression Pioglitazone modestly, but significantly, increased urine pH, the fraction of net acid excretion carried by NH^4+^(NH4+/NAE) and the ammoniagenic response (Δ NH^4+^/creatinine) after an acid load (Fig. 1). TZDs can reduce SUA levels (38). Pioglitazone also reduced net acid excretion and increased urine pH (5.37–5.59), the proportion of net acid excreted as ammonium, and ammonium excretion in response to an acute acid load (36).

### Glucagon-like peptide-1 receptor agonists

Glucagon-like peptide-1 receptor agonists (GLP-1 RAs) exert hypoglycemic effects by activating the GLP-1 receptor in a glucose concentration-dependent manner, stimulating insulin secretion and inhibiting glucagon secretion, while increasing glucose uptake in muscle and adipose tissue and inhibiting liver glucose production, gastric emptying, and appetite. In the USA, a survey reported that GLP-1 RA use increased by 8.5% in 2019 (39). SUA levels remained unchanged (Table 1) after 52 weeks of exenatide treatment (*n* = 36 patients with T2DM and overweight) (40). A similar outcome was observed in a *post* *hoc* analysis of four clinical trials (exenatide, lixisenatide, liraglutide, and reference group) (41). In contrast to previous findings, the analysis (42) showed that, based on 20 studies, GLP-1 RA might considerably lower the SUA levels by 0.34 mg/dL. Even when analyzed in subgroups of clinical trials and observational studies, the reductions remained significant. In T2DM patients and overweight healthy men, immediate exenatide infusion raised UE_UA_, most likely through blocking Na+/H+-exchanger type 3 in the renal proximal tubule (41). In addition, GLP-1 RA has been shown to suppress NHE3 activity (Fig. 1), a major route of fluid, NaCl, and bicarbonate reabsorption located in the apical membrane of the proximal tubule and thick ascending limb of Henle (43). Additionally, it improves cardiovascular health by lowering blood pressure and thus the risk of hypertension (44, 45). Accordingly, the administration of GLP-1 RAs can significantly reduce SUA levels (42).

### Dipeptidyl-peptidase 4 inhibitor

Dipeptidyl-peptidase 4 inhibitor (DPP-4i) reduces the inactivation of GLP-1 *in vivo* by inhibiting dipeptidyl peptidase IV, leading to an increase in endogenous GLP-1 levels. GLP-1 suppressed glucagon and stimulated insulin secretion in a concentration-dependent manner. Currently, there are four DPP-4i drugs that have been FDA-approved: sitagliptin, saxagliptin, linagliptin, and alogliptin (24). DPP-4i monotherapy accounted for 7.5% of the cases (24). Sitagliptin significantly increased SUA levels after 12 weeks (*n* = 940 patients with T2DM) (Table 1) (46). This result is identical to that of a previous study (*n* = 120 patients with T2DM) (47). Additional data on sitagliptin, alogliptin, and linagliptin are listed; the first two increased SUA levels, whereas the latter reduced SUA levels (48, 49). Xanthine oxidase (XO) is a well-established therapeutic target in hyperuricemia. It catalyzes the oxidation of inosine to hypoxanthine, xanthine, and uric acid (Fig. 1). Therefore, linagliptin reduces SUA levels in patients with T2DM (50). The mechanism of DPP-4i except linagliptin increasing SUA levels remains unclear. Under certain conditions, linagliptin is better than other DPP-4i medications for patients with T2DM and hyperuricemia.

### Sodium–glucose cotransporter 2 inhibitors

SGLT2i is a novel oral antidiabetic drug that has received considerable attention in recent years. It can reduce the renal glucose threshold, prevent the kidneys from reabsorbing glucose, and promote the excretion of sugar in the urine. In the USA, the use of SGLT2i has increased from 4.3% in 2015 to 18.5% in 2019 (39). SGLT2i also prevents the development of gout (51). Gout incidence was shown to be 64% lower in patients taking SGLT2i compared to those taking GLP-1 RA (4.9 vs 7.8 events/1000 patients/year) based on a U.S. nationwide insurance database encompassing 295,907 patients with T2DM (52). An interview revealed that empagliflozin significantly decreased SUA levels compared with placebo (*n* = 5781 patients with T2DM), among the SGLT2i class (Table 1) (53). Other experiments confirmed the same results on dapagliflozin (54, 55, 56, 57), canagliflozin (56, 57, 58, 59), and empagliflozin (56, 57). It is noteworthy that canagliflozin-induced SUA reduction may be affected by weight loss and baseline SUA levels. Canagliflozin significantly reduced SUA in the subgroup (*n* = 20 patients with T2DM and weight-loss) (from 5.75 ± 1.05 to 5.35 ± 1.17 mg/dL; *P* < 0.05) but not in the non-weight-losing group (from 5.18 ± 1.41 to 5.13 ± 1.27 mg/dL; P = ns) (58). In addition, subgroup (*n* = 40 patients with T2DM) receiving canagliflozin (50–100 mg) for 3 months showed a significant decrease in SUA (by 14.1%; *P* < 0.001) in those with higher baseline SUA (5.8 mg/dL), while those with lower baseline SUA (4.8 mg/dL) had a significant increase in SUA (by 11.4%; *P* < 0.001) (59). This physiological effect was reported that SGLT2i could preserve kidney function and subsequently increase uric acid excretion (through glycosuria and altered uric acid transport activity), thus reducing SUA levels (60, 61, 62, 63, 64). In particular, the SGLT2i-inducing increase in UEUA has been attributed to the inhibition of UA reabsorption by GLUT9b (Fig. 1) located in the collecting duct of the renal tubule (60, 65). For patients with T2DM with hyperuricemia, this medication is crucial. Based on previous experimental data and clinical experiences, SGLT2i is potentially advised for patients with T2DM and hyperuricemia.

### α-Glucosidase inhibitors

α-Glucosidase inhibitors are appropriate for patients with elevated postprandial (in diets that is primarily constituted of carbs) blood sugar because they lower postprandial blood sugar by preventing the upper section of the small intestine from absorbing carbohydrates. Owing to its ease of administration and high tolerance, it can be used as an adjunct to lifestyle modification for T2DM prevention (66). Glucosidase accounted for 35.9% in the OAD treatment. This effect was observed in 18% of monotherapies had this effect (67). One study (68) measured SUA and UEUA levels in six healthy participants (Table 1) before and after administering sucrose, with and without co-administration of acarbose. Sucrose increased the SUA levels by 10% (*P* < 0.01). However, the uric acid excretion and fractional clearance remained unaltered. Sucrose and acarbose co-administration also increased UEUA, but less than sucrose alone, without changes in urinary excretion and fractional clearance of uric acid (68). Sucrose is converted into fructose and glucose, which can increase SUA levels through increased purine degradation and/or decreased UEUA. By preventing sucrose absorption, acarbose can reduce the SUA increase caused by sucrose consumption (Fig. 1)(68, 69). In summary, acarbose alleviates the increase in the plasma concentration of uric acid induced by sucrose by inhibiting its absorption (68, 69).

### Sulfonylureas

Sulfonylureas are classified as insulin-secreting agents. Their primary pharmacological function was to increase the amount of insulin secreted by pancreatic islets β cells, hence lowering blood sugar and boosting insulin levels. Sulfonylureas constitute 7.7% of first-line therapies worldwide (24, 67). A study (70) (*n* = 29 patients with T2DM) with gliclazide reported no change in SUA levels. Similar results have been reported in another study (71). At present, the mechanism is unclear, and there is no evidence suggesting a connection between sulfonylureas and uric acid.

### Other drugs

There have been no relevant studies on the mechanisms or clinical data related to SUA levels for meglitinides and amylin analogs.

## Discussion

Notably, a part of patients with T2DM use more than two antidiabetic drugs to treat. Since the usage of in requires different renal functions which could affect SUA metabolism, we should conduct a more detailed and comprehensive analysis of such clinical results. It should be considered as a confounding factor in Table 1. Moreover, some studies (72, 73) suggest SUA levels decrease with increasing blood glucose levels. This is mainly attributed to the compensatory effect of the kidney via ultrafiltration. So in the listed data which come from all long-term studies, blood glucose is stable. Based on it, this compensatory effect will not affect the overall effect.

## Conclusion

Elevated SUA levels have been implicated in T2DM development and in CVD in patients with T2DM. SUA levels have been recognized as a predictor of DM development. Numerous pathways associated with lipid and glucose dysmetabolism, such as insulin resistance, oxidative stress, and inflammation, have been implicated in these relationships. Moreover, patients with DM with micro- or macrovascular complications had higher SUA levels than those without complications. SUA is the main component of renal stones in patients with T2DM. In light of these intricate and multifaceted interactions, further research are required to delineate the clinical implications of SUA measurements in the management and follow-up of patients with DM. In this literature review, we find that insulin and DPP-4i, with the exception of linagliptin, increases SUA levels. We think the usage of linagliptin and SGLT2i is the potentially effective treatment of patients with T2DM and hyperuricemia currently, while more RCT researches are required for a recommendation. Metformin, TZDs, GLP-1 RAs, and α-glucosidase inhibitors could decrease SUA. However, whether they can be used as potentially recommended drugs still requires further research for the treatment of patients with T2DM and hyperuricemia.

## Declaration of interest

The authors declare that there is no conflict of interest that could be perceived as prejudicing the impartiality of the this review.

## Funding

This review did not receive any specific grant from any funding agency in the public, commercial, or not-for-profit sector.

## Author contribution statement

Conceptualization: Baoyu Zhang, Zhenyu Liu; formal analysis and investigation: Zhenyu Liu, Huixi Kong; writing – original draft preparation: Zhenyu Liu; writing – review and editing: Zhenyu Liu; supervision: Baoyu Zhang.

## References

[1] Ong KL Stafford LK McLaughlin SA Boyko EJ Vollset SE Smith AE Dalton BE Duprey J Cruz JA Hagins H, *et al.* Global, regional, and national burden of diabetes from 1990 to 2021, with projections of prevalence to 2050: a systematic analysis for the global burden of disease study 2021. Lancet 2023 402 203–234. (10.1016/S0140-6736(2301301-6) 37356446 PMC10364581

[2] Tripathi BK & Srivastava AK. Diabetes mellitus: complications and therapeutics. Medical Science Monitor 2006 12 RA130–RA147. 16810145

[3] Chen L Magliano DJ & Zimmet PZ. The worldwide epidemiology of type 2 diabetes mellitus--present and future perspectives. Nature Reviews. Endocrinology 2011 8 228–236. (10.1038/nrendo.2011.183) 22064493

[4] GBD 2017 Diet Collaborators. Health effects of dietary risks in 195 countries, 1990–2017: a systematic analysis for the Global Burden of Disease Study 2017. Lancet 2019 393 1958–1972. (10.1016/S0140-6736(1930041-8) 30954305 PMC6899507

[5] Chen-Xu M Yokose C Rai SK Pillinger MH & Choi HK. Contemporary prevalence of gout and hyperuricemia in the United States and decadal trends: the national health and nutrition examination survey, 2007–2016. Arthritis and Rheumatology 2019 71 991–999. (10.1002/art.40807) 30618180 PMC6536335

[6] Zhang M Zhu X Wu J Huang Z Zhao Z Zhang X Xue Y Wan W Li C Zhang W, *et al.* Prevalence of hyperuricemia among Chinese adults: findings from two nationally representative cross-sectional surveys in 2015–16 and 2018–19. Frontiers in Immunology 2021 12 791983. (10.3389/fimmu.2021.791983) 35197964 PMC8858821

[7] Kuwabara M Kuwabara R Niwa K Hisatome I Smits G Roncal-Jimenez CA MacLean PS Yracheta JM Ohno M Lanaspa MA, *et al.* Different risk for hypertension, diabetes, dyslipidemia, and hyperuricemia according to level of body mass index in Japanese and American subjects. Nutrients 2018 10. (10.3390/nu10081011) PMC6115805 30081468

[8] Kim SK & Choe JY. Association between smoking and serum uric acid in Korean population: data from the seventh Korea national health and nutrition examination survey 2016. Medicine (Baltimore) 2019 98 e14507. (10.1097/MD.0000000000014507) 30762781 PMC6407981

[9] Jiang J Zhang T Liu Y Chang Q Zhao Y Guo C & Xia Y. Prevalence of diabetes in patients with hyperuricemia and gout: a systematic review and meta-analysis. Current Diabetes Reports 2023 23 103–117. (10.1007/s11892-023-01506-2) 37099085

[10] Shi H Liu Y Yang D Liang P Chen C Luan H & Shi C. Inverted U-shaped associations between serum uric acid and fasting - plasma glucose level in non-diabetic, pre-diabetic, and diabetic adults: a population-based study in China. Journal of Diabetes Investigation 2024 15 483–490. (10.1111/jdi.14132) 38108582 PMC10981146

[11] Kim SY Guevara JP Kim KM Choi HK Heitjan DF & Albert DA. Hyperuricemia and risk of stroke: a systematic review and meta-analysis. Arthritis and Rheumatism 2009 61 885–892. (10.1002/art.24612) 19565556 PMC2714267

[12] Ekundayo OJ Dell'Italia LJ Sanders PW Arnett D Aban I Love TE Filippatos G Anker SD Lloyd-Jones DM Bakris G, *et al.* Association between hyperuricemia and incident heart failure among older adults: a propensity-matched study. International Journal of Cardiology 2010 142 279–287. (10.1016/j.ijcard.2009.01.010) 19201041 PMC2906633

[13] Bos MJ Koudstaal PJ Hofman A Witteman JCM & Breteler MMB. Uric acid is a risk factor for myocardial infarction and stroke: the Rotterdam study. Stroke 2006 37 1503–1507. (10.1161/01.STR.0000221716.55088.d4) 16675740

[14] Zhao G Huang L Song M & Song Y. Baseline serum uric acid level as a predictor of cardiovascular disease related mortality and all-cause mortality: a meta-analysis of prospective studies. Atherosclerosis 2013 231 61–68. (10.1016/j.atherosclerosis.2013.08.023) 24125412

[15] Chatterjee S Khunti K & Davies MJ. Type 2 diabetes. Lancet 2017 389 2239–2251. (10.1016/S0140-6736(1730058-2) 28190580

[16] Zheng Y Ley SH & Hu FB. Global aetiology and epidemiology of type 2 diabetes mellitus and its complications. Nature Reviews. Endocrinology 2018 14 88–98. (10.1038/nrendo.2017.151) 29219149

[17] Cammalleri L & Malaguarnera M. Rasburicase represents a new tool for hyperuricemia in tumor lysis syndrome and in gout. International Journal of Medical Sciences 2007 4 83–93. (10.7150/ijms.4.83) 17396159 PMC1838823

[18] Jin M Yang F Yang I Yin Y Luo JJ Wang H & Yang XF. Uric acid, hyperuricemia and vascular diseases. Frontiers in Bioscience 2012 17 656–669. (10.2741/3950) PMC3247913 22201767

[19] Maiuolo J Oppedisano F Gratteri S Muscoli C & Mollace V. Regulation of uric acid metabolism and excretion. International Journal of Cardiology 2016 213 8–14. (10.1016/j.ijcard.2015.08.109) 26316329

[20] MacFarlane LA Liu CC & Solomon DH. The effect of initiating pharmacologic insulin on serum uric acid levels in patients with diabetes: a matched cohort analysis. Seminars in Arthritis and Rheumatism 2015 44 592–596. (10.1016/j.semarthrit.2014.10.008) 25455681 PMC4390413

[21] Mount DB Merriman T & Mandal A. Insulin: genetic and physiological influences on human uric acid homeostasis. Arthritis & Rheumatology 2018 70(suppl 9) abstract 2246. (available at: https://acrabstracts.org/abstract/insulin-genetic-and-physiological-influences-on-human-uric-acid-homeostasis/)

[22] Choi HK Mount DB Reginato AM, American College of Physicians & American Physiological Society. Pathogenesis of gout. Annals of Internal Medicine 2005 143 499–516. (10.7326/0003-4819-143-7-200510040-00009) 16204163

[23] Quiñones Galvan A Natali A Baldi S Frascerra S Sanna G Ciociaro D & Ferrannini E. Effect of insulin on uric acid excretion in humans. American Journal of Physiology 1995 268 E1–E5. (10.1152/ajpendo.1995.268.1.E1) 7840165

[24] Gomes MB Rathmann W Charbonnel B Khunti K Kosiborod M Nicolucci A Pocock SJ Shestakova MV Shimomura I Tang F, *et al.* Treatment of type 2 diabetes mellitus worldwide: baseline patient characteristics in the global DISCover study. Diabetes Research and Clinical Practice 2019 151 20–32. (10.1016/j.diabres.2019.03.024) 30904743

[25] Iliadis F Kadoglou NP Hatzitolios A Karamouzis M Alevizos M & Karamitsos D. Metabolic effects of rosiglitazone and metformin in Greek patients with recently diagnosed type 2 diabetes. In Vivo 2007 21 1107–1114. 18210765

[26] Gregorio F Ambrosi F Filipponi P Manfrini S & Testa I. Is metformin safe enough for ageing type 2 diabetic patients? Diabetes and Metabolism 1996 22 43–50. 8697295

[27] Barskova VG Eliseev MS Nasonov EL Volkov AV Tsapina TN Zilov AV Iakunina IA Il'inykh EV & Kudaeva FM. Use of metformin (siofor) in patients with gout and insulin resistance (pilot 6-month results). Terapevticheskiĭ Arkhiv 2005 77 44–49. 16514819

[28] Tetradze L Virsaladze D Javashvili L Kilasonia L & Tananashvili D. Relation of serum uric acid levels with basic metabolic parameters in patients with metabolic syndrome during insulin-sensitizing therapy. Georgian Medical News 2007 151 44–47. 18071212

[29] McCormick N O'Connor MJ Yokose C Merriman TR Mount DB Leong A & Choi HK. Assessing the causal relationships between insulin resistance and hyperuricemia and gout using bidirectional Mendelian randomization. Arthritis and Rheumatology 2021 73 2096–2104. (10.1002/art.41779) 33982892 PMC8568618

[30] Kimura Y Yanagida T Onda A Tsukui D Hosoyamada M & Kono H. Soluble uric acid promotes atherosclerosis via AMPK (AMP-activated protein kinase)-mediated inflammation. Arteriosclerosis, Thrombosis, and Vascular Biology 2020 40 570–582. (10.1161/ATVBAHA.119.313224) 31996020

[31] Yuan H Hu Y Zhu Y Zhang Y Luo C Li Z Wen T Zhuang W Zou J Hong L, *et al.* Metformin ameliorates high uric acid-induced insulin resistance in skeletal muscle cells. Molecular and Cellular Endocrinology 2017 443 138–145. (10.1016/j.mce.2016.12.025) 28042024

[32] Jiao Z Chen Y Xie Y Li Y & Li Z. Metformin protects against insulin resistance induced by high uric acid in cardiomyocytes via AMPK signalling pathways in vitro and in vivo. Journal of Cellular and Molecular Medicine 2021 25 6733–6745. (10.1111/jcmm.16677) 34053175 PMC8278091

[33] Society Chinese Diabetes & Zhu D. Guideline for the prevention and treatment of type 2 diabetes mellitus in China (2020 edition). Chinese Journal of Diabetes Mellitus 13 315–409. (10.3760/cma.j.cn311282-20210304-00142)

[34] Wilkinson S Douglas I Stirnadel-Farrant H Fogarty D Pokrajac A Smeeth L & Tomlinson L. Changing use of antidiabetic drugs in the UK: trends in prescribing 2000–2017. BMJ Open 2018 8 e022768. (10.1136/bmjopen-2018-022768) PMC6067400 30056393

[35] Kutoh E & Hori T. Effect of pioglitazone on serum uric acid levels in newly diagnosed, drug-naïve patients with type 2 diabetes. Endocrine Research 2013 38 151–159. (10.3109/07435800.2012.745128) 23216460

[36] Maalouf NM Poindexter JR Adams-Huet B Moe OW & Sakhaee K. Increased production and reduced urinary buffering of acid in uric acid stone formers is ameliorated by pioglitazone. Kidney International 2019 95 1262–1268. (10.1016/j.kint.2018.11.024) 30795852 PMC6478507

[37] Bobulescu IA Dubree M Zhang J McLeroy P & Moe OW. Reduction of renal triglyceride accumulation: effects on proximal tubule Na+/H+ exchange and urinary acidification. American Journal of Physiology. Renal Physiology 2009 297 F1419–F1426. (10.1152/ajprenal.00177.2009) 19692486 PMC2781342

[38] Rizos CV Liberopoulos EN Mikhailidis DP & Elisaf MS. Pleiotropic effects of thiazolidinediones. Expert Opinion on Pharmacotherapy 2008 9 1087–1108. (10.1517/14656566.9.7.1087) 18422468

[39] Heyward J Christopher J Sarkar S Shin JI Kalyani RR & Alexander GC. Ambulatory noninsulin treatment of type 2 diabetes mellitus in the United States, 2015 to 2019. Diabetes, Obesity and Metabolism 2021 23 1843–1850. (10.1111/dom.14408) 33881795

[40] Muskiet MHA Bunck MC Heine RJ Cornér A Yki-Järvinen H Eliasson B Joles JA Diamant M Tonneijck L & van Raalte DH. Exenatide twice-daily does not affect renal function or albuminuria compared to titrated insulin glargine in patients with type 2 diabetes mellitus: a post-hoc analysis of a 52-week randomised trial. Diabetes Research and Clinical Practice 2019 153 14–22. (10.1016/j.diabres.2019.05.001) 31078666

[41] Tonneijck L Muskiet MHA Smits MM Bjornstad P Kramer MHH Diamant M Hoorn EJ Joles JA & van Raalte DH. Effect of immediate and prolonged GLP-1 receptor agonist administration on uric acid and kidney clearance: post-hoc analyses of four clinical trials. Diabetes, Obesity and Metabolism 2018 20 1235–1245. (10.1111/dom.13223) PMC5899927 29341461

[42] Najafi S Bahrami M Butler AE & Sahebkar A. The effect of glucagon-like peptide-1 receptor agonists on serum uric acid concentration: a systematic review and meta-analysis. British Journal of Clinical Pharmacology 2022 88 3627–3637. (10.1111/bcp.15344) 35384008

[43] Li HC Du Z Barone S Rubera I McDonough AA Tauc M Zahedi K Wang T & Soleimani M. Proximal tubule specific knockout of the Na⁺/H⁺ exchanger NHE3: effects on bicarbonate absorption and ammonium excretion. Journal of Molecular Medicine 2013 91 951–963. (10.1007/s00109-013-1015-3) 23508938 PMC3730089

[44] Martins FL Bailey MA & Girardi ACC. Endogenous activation of glucagon-like peptide-1 receptor contributes to blood pressure control: role of proximal tubule Na+/H+ exchanger isoform 3, renal angiotensin II, and insulin sensitivity. Hypertension 2020 76 839–848. (10.1161/HYPERTENSIONAHA.120.14868) 32755467

[45] Drucker DJ. The cardiovascular biology of glucagon-like peptide-1. Cell Metabolism 2016 24 15–30. (10.1016/j.cmet.2016.06.009) 27345422

[46] Kubota A Maeda H Kanamori A Matoba K Jin Y Minagawa F Obana M Iemitsu K Ito S Amemiya H, *et al.* Pleiotropic effects of sitagliptin in the treatment of type 2 diabetes mellitus patients. Journal of Clinical Medicine Research 2012 4 309–313. (10.4021/jocmr1061w) 23024732 PMC3449427

[47] Matsushima Y Takeshita Y Kita Y Otoda T Kato K-I Toyama-Wakakuri H Akahori H Shimizu A Hamaguchi E Nishimura Y, *et al.* Pleiotropic effects of sitagliptin versus voglibose in patients with type 2 diabetes inadequately controlled via diet and/or a single oral antihyperglycemic agent: a multicenter, randomized trial. BMJ Open Diabetes Research and Care 2016 4 e000190. (10.1136/bmjdrc-2015-000190) PMC4838664 27110370

[48] Kutoh E Wada A & Hayashi J. Regulation of free fatty acid by sitagliptin monotherapy in drug-naïve subjects with type 2 diabetes. Endocrine Practice 2018 24 1063–1072. (10.4158/EP-2018-0287) 30289315

[49] Tojikubo M & Tajiri Y. Different effects of linagliptin and sitagliptin on blood pressure and renal function in Japanese patients with type 2 diabetes mellitus. Diabetology International 2017 8 397–401. (10.1007/s13340-017-0320-4) 30603346 PMC6224920

[50] Yamagishi S-i Ishibashi Y Ojima A Sugiura T & Matsui T. Linagliptin, a xanthine-based dipeptidyl peptidase-4 inhibitor, decreases serum uric acid levels in type 2 diabetic patients partly by suppressing xanthine oxidase activity. International Journal of Cardiology 2014 176 550–552. (10.1016/j.ijcard.2014.07.023) 25065332

[51] Sheu WHH. Lowering the risk of gout: another benefits from the use of sodium-glucose cotransporter 2 inhibitors. Journal of Diabetes Investigation 2020 11 1115–1116. (10.1111/jdi.13254) 32190971 PMC7477493

[52] Fralick M Chen SK Patorno E & Kim SC. Assessing the risk for gout with sodium-glucose cotransporter-2 inhibitors in patients with type 2 diabetes: a population-based cohort study. Annals of Internal Medicine 2020 172 186–194. (10.7326/M19-2610) 31931526 PMC7217750

[53] Zhao D Liu H & Dong P. Empagliflozin reduces blood pressure and uric acid in patients with type 2 diabetes mellitus: a systematic review and meta-analysis. Journal of Human Hypertension 2019 33 327–339. (10.1038/s41371-018-0134-2) 30443007

[54] Hao Z Huang X Shao H & Tian F. Effects of dapagliflozin on serum uric acid levels in hospitalized type 2 diabetic patients with inadequate glycemic control: a randomized controlled trial. Therapeutics and Clinical Risk Management 2018 14 2407–2413. (10.2147/TCRM.S186347) 30587997 PMC6294165

[55] Fuchigami A Shigiyama F Kitazawa T Okada Y Ichijo T Higa M Hiyoshi T Inoue I Iso K Yoshii H, *et al.* Efficacy of dapagliflozin versus sitagliptin on cardiometabolic risk factors in Japanese patients with type 2 diabetes: a prospective, randomized study (DIVERSITY-CVR). Cardiovascular Diabetology 2020 19 1. (10.1186/s12933-019-0977-z) 31910850 PMC6945792

[56] Zhao Y Xu L Tian D Xia P Zheng H Wang L & Chen L. Effects of sodium-glucose co-transporter 2 (SGLT2) inhibitors on serum uric acid level: a meta-analysis of randomized controlled trials. Diabetes, Obesity and Metabolism 2018 20 458–462. (10.1111/dom.13101) 28846182

[57] Xin Y Guo Y Li Y Ma Y Li L & Jiang H. Effects of sodium glucose cotransporter-2 inhibitors on serum uric acid in type 2 diabetes mellitus: a systematic review with an indirect comparison meta-analysis. Saudi Journal of Biological Sciences 2019 26 421–426. (10.1016/j.sjbs.2018.11.013) 31485187 PMC6717127

[58] Kutoh E Wada A Murayama T & Hayashi J. Two glucose-lowering mechanisms of canagliflozin depending on body weight changes in drug-naïve subjects with type 2 diabetes. Drugs in R&D 2018 18 309–315. (10.1007/s40268-018-0250-z) 30324549 PMC6277318

[59] Kutoh E Wada A Kuto AN & Hayashi J. Regulation of serum uric acid with canagliflozin monotherapy in type 2 diabetes: A potential link between uric acid and pancreatic β-cell function. International Journal of Clinical Pharmacology and Therapeutics 2019 57 590–595. (10.5414/CP203513) 31587751

[60] Bailey CJ. Uric acid and the cardio-renal effects of SGLT2 inhibitors. Diabetes, Obesity and Metabolism 2019 21 1291–1298. (10.1111/dom.13670) 30762288

[61] Chino Y Samukawa Y Sakai S Nakai Y Yamaguchi J-i Nakanishi T & Tamai I. SGLT2 inhibitor lowers serum uric acid through alteration of uric acid transport activity in renal tubule by increased glycosuria. Biopharmaceutics and Drug Disposition 2014 35 391–404. (10.1002/bdd.1909) 25044127 PMC4223977

[62] Yaribeygi H Butler AE Atkin SL Katsiki N & Sahebkar A. Sodium-glucose cotransporter 2 inhibitors and inflammation in chronic kidney disease: possible molecular pathways. Journal of Cellular Physiology 2018 234 223–230. (10.1002/jcp.26851) 30076706

[63] Katsiki N Mikhailidis DP & Theodorakis MJ. Sodium-glucose cotransporter 2 inhibitors (SGLT2i): their role in cardiometabolic risk management. Current Pharmaceutical Design 2017 23 1522–1532. (10.2174/1381612823666170113152742) 28088910

[64] Katsiki N Papanas N & Mikhailidis DP. Dapagliflozin: more than just another oral glucose-lowering agent? Expert Opinion on Investigational Drugs 2010 19 1581–1589. (10.1517/13543784.2011.539558) 21105857

[65] Ahmadieh H & Azar S. Effects of sodium glucose Cotransporter-2 inhibitors on serum uric acid in type 2 diabetes mellitus. Diabetes Technology and Therapeutics 2017 19 507–512. (10.1089/dia.2017.0070) 28749169

[66] Dahlén AD Dashi G Maslov I Attwood MM Jonsson J Trukhan V & Schiöth HB. Trends in antidiabetic drug discovery: FDA approved drugs, new drugs in clinical trials and global sales. Frontiers in Pharmacology 2021 12 807548. (10.3389/fphar.2021.807548) 35126141 PMC8807560

[67] Ji L Lu J Weng J Jia W Tian H Zhu D Xing X & Guo L. China type 2 diabetes treatment status survey of treatment pattern of oral drugs users. Journal of Diabetes 2015 7 166–173. (10.1111/1753-0407.12165) 24809622

[68] Moriwaki Y Inokuchi T Ka T Yamamoto A Tsutsumi Z Takahashi S & Yamamoto T. Effect of acarbose on the increased plasma concentration of uric acid induced by sucrose ingestion. Nucleosides, Nucleotides and Nucleic Acids 2008 27 631–633. (10.1080/15257770802138699) 18600518

[69] Moriwaki Y Kobayashi T Inokuchi T Yamamoto A Takahashi S Ka T Tsutsumi Z & Yamamoto T. Acarbose alleviates rise in plasma uric acid concentration induced by sucrose ingestion. International Journal of Clinical Pharmacology and Therapeutics 2008 46 187–192. (10.5414/cpp46187) 18397692

[70] Kilo C Dudley J & Kalb B. Evaluation of the efficacy and safety of Diamicron in non-insulin-dependent diabetic patients. Diabetes Research and Clinical Practice 1991 14(Supplement 2) S79–S82. (10.1016/0168-8227(9190012-3) 1794270

[71] Diwan V Gobe G & Brown L. Glibenclamide improves kidney and heart structure and function in the adenine-diet model of chronic kidney disease. Pharmacological Research 2014 79 104–110. (10.1016/j.phrs.2013.11.007) 24291534

[72] Li H Zha X Zhu Y Liu M Guo R & Wen Y. An invert U-shaped curve: relationship between fasting plasma glucose and serum uric acid concentration in a large health check-up population in China. Medicine (Baltimore) 2016 95 e3456. (10.1097/MD.0000000000003456) 27100447 PMC4845851

[73] Wang Y Chi J Che K Chen Y Sun X Wang Y & Wang Z. Fasting plasma glucose and serum uric acid levels in a general Chinese population with normal glucose tolerance: a U-shaped curve. PLoS One 2017 12 e0180111. (10.1371/journal.pone.0180111) 28658284 PMC5489204

